# Strobe sequence design for haplotype assembly

**DOI:** 10.1186/1471-2105-12-S1-S24

**Published:** 2011-02-15

**Authors:** Christine Lo, Ali Bashir, Vikas Bansal, Vineet Bafna

**Affiliations:** 1 Department of Computer Science and Engineering, University of California, San Diego, La Jolla, CA 92093, USA; 2 Pacific Biosciences, 1505 Adams Drive, Menlo Park, CA 94025, USA; 3 Scripps Genomic Medicine, Scripps Translational Science Institute, La Jolla, CA 92037, USA

## Abstract

**Background:**

Humans are diploid, carrying two copies of each chromosome, one from each parent. Separating the paternal and maternal chromosomes is an important component of genetic analyses such as determining genetic association, inferring evolutionary scenarios, computing recombination rates, and detecting *cis*-regulatory events. As the pair of chromosomes are mostly identical to each other, linking together of alleles at heterozygous sites is sufficient to phase, or separate the two chromosomes. In Haplotype Assembly, the linking is done by sequenced fragments that overlap two heterozygous sites. While there has been a lot of research on correcting errors to achieve accurate haplotypes via assembly, relatively little work has been done on designing sequencing experiments to get long haplotypes. Here, we describe the different design parameters that can be adjusted with next generation and upcoming sequencing technologies, and study the impact of design choice on the length of the haplotype.

**Results:**

We show that a number of parameters influence haplotype length, with the most significant one being the advance length (distance between two fragments of a clone). Given technologies like strobe sequencing that allow for large variations in advance lengths, we design and implement a simulated annealing algorithm to sample a large space of distributions over advance-lengths. Extensive simulations on individual genomic sequences suggest that a non-trivial distribution over advance lengths results a 1-2 order of magnitude improvement in median haplotype length.

**Conclusions:**

Our results suggest that haplotyping of large, biologically important genomic regions is feasible with current technologies.

## Background

**Figure 1:**
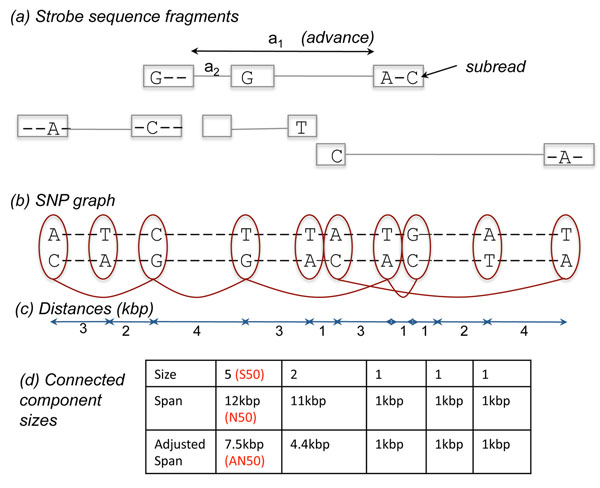
**Schematic for Haplotype Assembly.** (a) Strobe sequencing allows for the generation of subreeds, separated by user-defined advance lengths. The reads can be mapped to the reference to detect heterozygous sites. (b) Nodes in the SNP-Graph correspond to heterozygous sites. Edges correspond to pairs of sites that are linked by a fragment. Haplotype assembly is limited to connected components of the SNP-Graph. (c) The distance between sites is used for measuring haplotype lengths. (d) The S50, N50, and AN50 measure of haplotype assembly.

Humans are diploid, inheriting a pair of each chromosome, one from each parent. The two copies of each chromosome are highly homologous to each other. With most current technologies, heterozygous sites are sampled independently from both chromosomes, and the data appears as a collection of heterozygous sites. See Figure 1b. The goal of *haplotype phasing* is to separate the maternal and paternal chromosomes, by linking alleles at heterozygous sites.

Haplotyping is an important component of genetic analysis. It improves the power of genetic association, and is useful in inferring evolutionary scenarios, historical recombination events, and detecting *cis*-regulatory events [1,2]. Given the importance of the problem, a variety of computational and experimental techniques have been developed to phase chromosomes, and we discuss a few here to put our work in context. *Population-based inference* exploits linkage disequilibrium to identify likely phasings. Consider a population of individuals sampled at the first two sites in Figure 1. If a large number of individuals carry the homozygous genotypes (A/A-T/T), then we infer that the haplotype AT is common in the population, and the phasing (AT,CA). However, historical recombination events can reduce or eliminate this linkage, and reliable phasing can only be achieved over short regions, 3050Kb on the average [3,4]. While phasing is difficult with populations, it is almost trivial if parental information is known [2,5]. In *family-based haplotyping*, if the mother and the father of the individual in Figure 1 had genotypes (A/A-T/A) and (A/C-A/A), then the individual shows (A/C-T/A) only by inheriting AT from the mother and CA from the father. Given that only a few crossovers occur per meiosis on each chromosome, a small sampling of homozygous alleles in the parents is sufficient to phase entire chromosomes. While family-based haplotyping is powerful, it is not always feasible and requires additional genotyping or sequencing of parents. Also, family-based techniques will not work for haplotyping in a more general context, where it can also refer to separating strains of microbes and pooled samples from other organisms. Recently, *chromosome micro-dissection* techniques have also been developed to amplify genomic DNA from single molecule templates. The principle is as follows: micro-dissect and dilute DNA, so that each sample contains only one chromosomal fragment; perform whole genome amplification, followed by genotyping/sequencing. Homozygous sequenced alleles originate from a single chromosome and can be linked [6]. While this method works for connecting distal heterozygous sites, current techniques provide only a very sparse phasing-about 24K heterozygous SNPs in Ma et al., 2010 [6]. The approach must be used in conjunction with other techniques to get meaningful results.

The method we discuss here, *Haplotype Assembly*, is very attractive given the proliferation of inexpensive sequencing techniques [7,8] that have the throughput to sequencing entire human genomes.See Figure 1a-b. Each sequenced fragment is sampled from one of the chromosomes and mapped to the reference sequence. Multiple alleles sampled by one fragment must all be from the same chromosome. Therefore, the fragment -A-C- links allele A (site 1) with allele C (site 3). If a sufficiently large number of informative fragments (linking heterozygous sites) are available, long haplotypes can be generated by chaining the links together. Haplotype assembly was proposed some time ago [9,10], but the data for individual genomes is only now becoming available. The first sequence of a genomic individual, J. Craig Venter, (designated, *HuRef)* was produced using Sanger sequencing. The sequencing was paired-end, and we modify notation slightly to say that Sanger sequencing generated ~2000 bp per *read,* with two *subreads* linked 2kbp-150kbp apart, and each base sampled an average of 6×. This change of notation allows us to discuss strobe sequencing later, where each read can have arbitrary *k* ≥ 1 subreads. The phasing was quite effective, with a ‘median’ haplotype (the metric is precisely defined later) of length 270kbp [11]. Specialized error correction algorithms were used to generate highly accurate haplotypes [12,13]. Sanger sequencing provides long and accurate reads but lower throughput and expensive library preparation making it less cost-effective. By contrast, newer technologies allow for massively parallel sequencing, but have much shorter reads, and are more error prone. While there has been ongoing work on haplotype assembly [14], much of it has focused on one aspect of the problem, as explained below.

We begin by formalizing the problem. Aligned fragments define a *SNP-Graph* in the individual, as shown in Figure 1b. Each heterozygous location corresponds to a node. When a fragment overlaps two sites, we add an edge to the corresponding nodes. It is easy to see that two sites can be phased if and only if they are connected in the SNP-Graph. Therefore the *length* of the haplotypes depend upon the size of connected components, while the *accuracy* of haplotypes depends upon the error in sequencing, depth of coverage, and computational algorithms for error correction. The quality of a haplotype is measured by metrics for length and accuracy.

### Metrics for haplotype length

Given the SNP-Graph, we use three different metrics (S50, N50, AN50) to measure the median length of assembled haplotypes: S50, N50, and AN50, related to the size (number of SNPs), span (distance spanned), and adjusted span of the contigs respectively. See Figure 1d. Recall that the haplotyping is limited to connected components in the SNP-Graph. The length of a haplotype can be described in terms of its *size* (# of heterozygous sites), or span (distance between distal heterozygous sites). As the connected components can interleave, we define the *adjusted-span* of a component as the span times the fraction of sites that lie in the contig. In Figure 1d, we observed connected components of size 5 and 2 with spans 12kbp, and 11kbp, respectively. The adjusted spans are given by, , and .

We define *S*50 (and N50) to be the *size* (respectively, span) such that 50% of all sites are in contigs of size (span) *S*50 (N50), or greater. As SNPs display a ‘clumping’ property, S50 might inflate the haplotype size. On the other hand, N50 tends to inflate the haplotype size when there are contigs that span a long distance, but do not phase many SNPs. The AN50, or adjusted N50 metric considers both span, and size. It is defined as the adjusted span s.t. 50% of the SNPs are in contigs with an adjusted span AN50 or larger. We will primarily use the AN50 metric. However, our results and trends remain the same for any metric.

### Metrics for haplotype accuracy

Erroneous base-calls corrupt the accuracy of assembled haplotypes. In simulations, where the reference is known, we can measure the accuracy of the reconstructed haplotype as the *haplotype edit rate (HER)*, equal to the fraction of incorrectly called alleles. A second reason for incorrect haplotyping is that weak links might cause a ‘switch’, a crossover from one true haplotype to the other. This could potentially cause HER to be large, even though a single crossover can correct the haplotypes. See Additional File 1. Therefore, we define another metric *switch error rate (SER)* which is the number of crossovers (per heterozygous site) in the assembled haplotypes to match the correct haplotype.

### Strobe sequencing and haplotype assembly

Much of the current computational research on haplotype assembly focuses on improving haplotype accuracy [12-14]. Until now, the length of the haplotypes depended upon the specific technological parameters, and was assumed to be determined by the technology. With recent developments in sequencing, the user has the ability to select different parameters for an experiment. Our paper investigates the relationship of sequencing parameters on the haplotype length.

Of particular relevance is the upcoming technology of *strobe sequencing,* available from Pacific Bio-sciences [15]. In this technology, a genomic fragment is sequenced in a *strobed fashion* with sub-reads of pre-determined lengths separated by user-determined intervals *(advances).* In Figure 1a, we see a number of fragments with *k* = 2 strobes, and one with 3 strobes. Paired-end sequencing is analogous to strobe sequencing with *k* = 2, however it differs in that the sequenced reads must be from terminal portions of an insert which leads to reduced flexibility in selecting advance lengths. A key result of our analysis is that the choice of advance lengths can change the haplotype length by an order of magnitude for the same amount of sequencing. In fact, the best results are obtained by a complex distribution *f* on advance lengths. Besides *k* and *f* we also study the impact of other parameters on haplotype length. These include (a) L, the number of bp sequenced per fragment; *L* = ∑*_i_l_i_*, where *l_i_* is the length of the *i*-th subread; (b) *N*: number of fragments sequenced; (c) *A*, the maximum insert size allowed. Note that because we usually fix L, the advance lengths are related to *A.* For example, the maximum advance length for *k* = 2 strobes is *A* – *L*. In addition, we usually work with coverage *c* = *NL*/*G*, which gives the number of times each bp is sampled, on average. To obtain our results, we developed a simulator that generates reads according to specific technological parameters, and constructs connected components of the SNP-Graph. The software is available upon request from the authors.

While the focus of our analysis is on designing experiments for haplotype length, we also touch upon haplotype accuracy. We use a simulator provided by Pacific Biosciences to generate strobe sequence data based on an error model having high rates (roughly symmetric) of insertions and deletions relative to miscall errors [16]. We use our previously designed tools to phase in the presence of error. Our results indicate that long and accurate haplotyping is feasible even with technology having such high error rates.

## Results and discussion

### Singleton strobes

**Figure 2:**
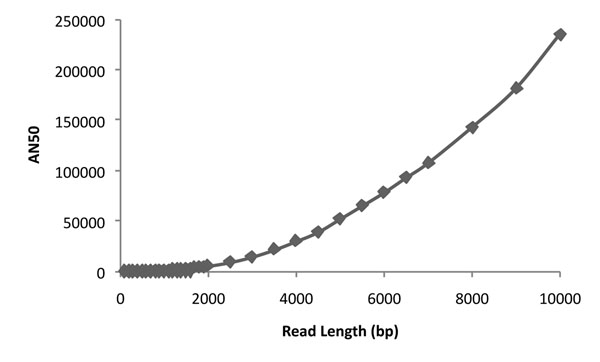
**Haplotype Assembly with Singleton Strobe.** (a) Distribution of Inter-SNP Distances. The log-log plot suggests slower than exponential decay, better fit by a power-law. (b) With single strobes (*k* = 1), high coverage and very long reads are needed to achieve significant haplotypes.

Assuming that the cost is proportional to the number of nucleotides sequenced, we compare all designs after fixing coverage c. A back-of-the-envelope calculation suggests that with long read lengths (*L* ≃ 1kbp), we should be able to link all SNPs together, given that the average pair of SNPs is 1kbp apart. The intuition is wrong because (a) a Poisson process for SNPs implies an exponential distribution of inter-SNP distance in a population- hence a long tail; and, (b) a single individual is heterozygous at only a subset of the SNPs. Indeed, the distribution of inter-SNP distances in HuRef is more consistent with the power-law (than exponential) with a long tail of large Inter-SNP distances (Figure 2a). Therefore, we only reach an AN50=48kbp even with *L* = 5kbp and *c* = 20× (Figure 2b). Similar results can be obtained with mate pair sequencing (*k* = 2) at much lower coverage. The linking together of distant SNPs through subread probes is indeed the most significant parameter determining haplotype length.

### Advance Lengths for Paired End Sequencing (*k* = 2)

We fixed the read-length *L* = 900bp as it is within the current mean length distribution reported by Pacific Biosciences [17]. For *L* = 900bp, *c* = 20×, and *k* = 2 subreads, choosing fixed insert sizes *A*_1_ = 3kbp, *A*_2_ = 9kbp results in low AN50 values 5.4kbp and 6.7kbp, respectively. However, a simple 50-50 mix of the two increases this by an order of magnitude AN50=54kbp. Clearly, variation in insert size, and thus advance length, is important. However, it is not immediately obvious what distribution of advance lengths will give the highest AN50. For example, we could consider uniformly varying advances from a minimum to a maximum length, or follow the library mix used for sequence assembly dominated by smaller advance lengths to form contigs, mixed with a smaller number of large advances to create scaffolds. To search efficiently over a large space of distributions, we used the 2-parameter *β-*distribution. For parameters (*α*, *β*), and maximum insert size *A*, define the p.d.f as(1)

where the denominator is a normalizing constant. Different choices of *α*, *β* provide a large range of distributions for *f*(*a*) [18]. For example, larger *α* values correspond to a negative skew (longer advance lengths are preferred), while larger *β* correspond to a more positive skew. When *α* = *β,* the distribution is symmetric. We systematically explored all *α, β* values in the interval (0 – 4]. Additionally, we implemented a simulated annealing algorithm (Methods) to identify the optimal choice of parameters.

**Figure 3:**
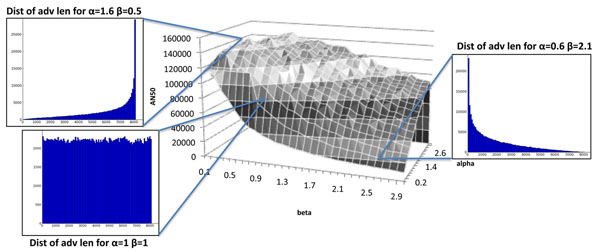
**AN50 for various *α*, *β* values.** This grid was created by simulating the first 10Mbp of chr1 (HuRef), at *L* = 900bp, *c* = 20×, *A* = 9kbp, *α* ∈ (0,3.4] and *β* ∈ (0,3]. Each (*α*, *β*) pair was simulated 25 times, and the median AN50 was plotted.

Surprisingly, the distributions with the highest AN50 had *α* ∈ [1.0 – 3.2] and *β* ∈ [0.3 – 0.9], and skewed heavily toward the longer clones. For *c* = 20×, L = 900bp, *A* = 9kbp, (*α*, *β*) = (1.6, 0.5), we achieve an AN50≃ 151kbp (Figure 3). Even more surprising, distributions skewed toward smaller clone lengths (*α*, *β*) = (0.6, 2.3) had the worst performance (AN50=38kbp). Uniform (*α*, *β*) = (1,1), and other symmetric distributions (*α* = *β*) show an intermediate performance. The bias is maintained at different values of coverage, maximum insert size, and other parameters. While there is a heavy bias towards longer clones, variation is important as well. For example, the distribution given by (*α*, *β*) = (4.5, 0.1) shows an extreme skew towards longer clone lengths so that it almost mimics a delta function at 9kbp and gives an AN50 of 45kbp. The trends do not change with a choice of other metrics S50, N50 (see Additional File 2b-d).

**Figure 4:**
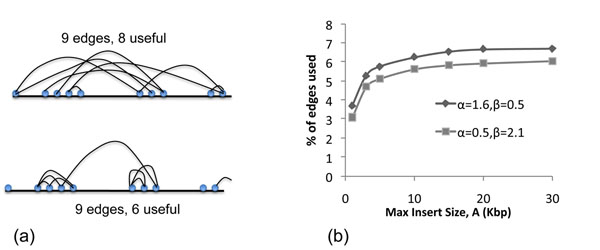
**Increase in the number of useful edges with increasing *A.*** (a) A schematic illustration of the impact of designs on the number of useful edges. As there is a larger number of SNP pairs with small distances, designs that favor short edges tend to connect already connected components, and have less useful edges. (b) The fraction of useful edges for two designs, and for a range of values for *A.* Note that a large fraction of edges (reads) is wasted either because the edges do not connect two variants, or because the they connect already connected components. The design with longer advance lengths always has a larger fraction of useful edges resulting in better AN50. Simulations used *L* = 900bp, *c* = 20×, *A* = 9kbp.

**Wasted reads:** Note that popular designs for sequence assembly emphasize short inserts (with a tight distribution of insert-lengths) mixed with a few large clones for scaffolding. By contrast, haplotype assembly is improved by focusing on larger inserts and higher variation. Figure 4a provides an illustration of the impact of different distributions of advance lengths on the connectivity of the SNP-Graph. A connected component with *k* vertices and *m* edges has *m* – *k* + 1 ‘waste’ edges, as only *k* – 1 ‘useful’ edges are needed to maintain connectivity. Due to the clustering of SNPs, a design with larger number of short advances has more wasted edges compared to a design with long advances. As each useful edge connects two previously unconnected components, it has a large impact on haplotype lengths. We computed the number of useful edges for the two designs, fixing *c* = 20× and varying maximum insert, *A.* We observe that the number of useful edges is always larger in designs with a bias towards long advance lengths (Figure 4b). For *A* = 5kbp, we see a 13% difference in useful edges between the two distributions.

The Erdós-Renyi theory describes the evolution of a random graph from isolated components to a single component, with increasing number of edges [19]. In our case, the edge probability in SNP-graphs is not initially uniform due to the clustering of SNPs (i.e. there is a bias towards proximal SNP pairs). By choosing designs with a bias towards longer advance lengths, we are essentially leveling out the probability of linking SNP pairs irrespective of their distance, leading to improved connectivity.

### Other parameters

**Figure 5:**
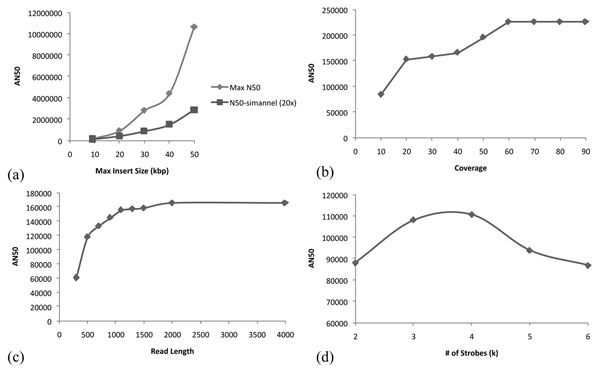
**AN50 for Other Parameters.** (a) Increasing *A* increases AN50. (b) Increasing *c* increases AN50 until saturation. (c) Increasing *L* increases AN50, but saturates quickly. (d) Using more strobes increases the variation of advance lengths, but decreases the size of the subreads. AN50 is maximized at *k* = 3,4. All simulations were performed on the first 10Mbp of chr1 (HuRef) using *L* = 900bp, *c* = 20×, *A* = 9kbp. SA based optimal (*α*, *β*) values were similar, and produced advance length distributions skewed towards longer advances. (See Additional File 3 for exact (*α*, *β*) values.)

**Maximum Insert Size,***A***:** In Figure 5a, we plot maximum achieved AN50 (for *c* = 20×, *L* = 900bp) maximum theoretical AN50 (assuming infinite coverage) as a function of *A.* The achieved AN50 increases with increasing *A* for the same amount of sequencing (c = 20×), indicating that the largest possible value of *A* should be chosen. Interestingly, the SA optimized parameters (*α*, *β*) remain similar as *A* is increased (See Additional File 1).

**Coverage, *c*:** The effect of coverage on AN50 is analogous to increasing the edge probability, and we expect to see an increase in connectivity until saturation is reached. The plot in Figure 5b shows this for *A* = 9000bp, *L* = 900bp, and SA optimized (*α*, *β*)*.*

**Read length, *L:*** Once *A*, *c* are fixed the impact of read-length *L* is minimal. Here, we assume that the subread is of minimal size (≥ 100) to permit accurate mapping. Initial improvement is seen with increasing *L* as the same subread captures proximal SNPs. However, the effect saturates quickly. (Figure 5c shows this for *A* = 9kbp,*k* = 2, *c* = 20× and SA optimized (*α*, *β*) values. Again, the *β*-distribution stays similar with changes in *L.* (See Additional File 1)

### Number of strobes, *k*

Besides flexibility in advance lengths, strobe sequencing allows the possibility of multiple strobes *k.* Figure 1a provides a cartoon of strobe sequencing for *k* = 2 and *k* = 3. To compare designs with different number of strobes, we fixed the subread lengths for each *k* to *l_k_* = *L*/*k*, keeping the total read-length constant. We also fixed the maximum insert size, *A*. Recall from the paired-end results that longer sub-read lengths help cover the relatively high proportion of SNPs that are clustered close together. Therefore, increasing number of strobes helps increase the variation in advance lengths against the penalty of smaller subreads.

### Optimal advance distribution for higher *k*

For a simulation with *k* strobes, we compute an optimal collection of (*α_i_*, *β_i_*) for 0 <*i* <*k* iteratively. Thus, for *k* = 3, *a*_1_ is randomly generated with (*α*_1_*, β*_1_*, A*)*,* and a_2_ is randomly generated with (*α*_2_, *β*_2_, *a*_1_). The strobed read is arranged as in Figure 1a with *a*_1_ as the advance length between the subread_1_ and subread_3_, and *a*_2_ as the advance length between subread_1_ and subread_2_. A similar pattern is used for higher *k*. While we see an improvement for *k* = 3 and *k* = 4, higher values of *k* do not help (Figure 5d).

The optimal distribution always skewed towards longer advance lengths. The skew towards longer advance lengths was extremely strong, and consistent among the very first set of (*α*, *β*)’s chosen, corresponding to the advance length between to two furthest strobes. For the other set of (*α*,*β*)’s, there was still a skew towards the longer advance lengths; however, the skew was not as strong and the degree of the skew was much more varied. We conclude that for the shorter advance lengths among multiple strobes, the *exact* distribution does not have a strong effect, as long as it is skewed towards longer advance lengths.

### Regions with a high SNP density

**Figure 6:**
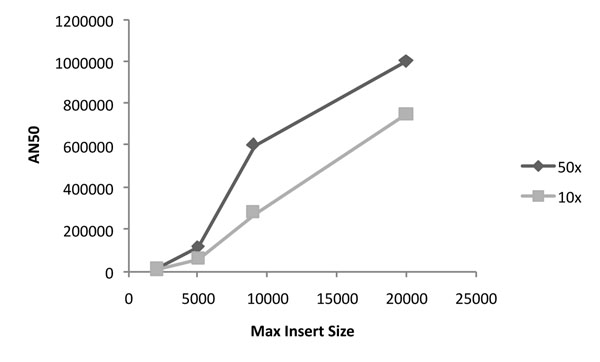
**Haplotyping the HLA Region** (chr6:29,652K-33,130K) of HuRef. AN50 increases with maximum insert, and increased coverage. For *c* = 10, *A* = 20K, 5 haplotypes cover 80% of the 3.5Mbp region.

Haplotype assembly is often applied to phase specific regions of interest. Often, these regions are gene-rich, and have a high SNP density. The HLA Region on chromosome 6, contains genes encoding cell surface antigen presenting genes and many other genes involved in the immune system. Diversity in this region is important for host defense against pathogens, and it has been implicated in susceptibility to diseases including diabetes, cancer, and various autoimmune disorders [20,21]. Phasing of coding SNPs could provide critical structural information, motivating the development of haplotyping techniques specifically targeted to this region [22]. We specifically looked at the region from position chr6:29, 652K-33,130K, using HuRef data. While increased coverage provides modest improvement, high gains in AN50 are obtained by increasing *A* (Figure 6). At *c* = 10×, *L* = 900bp, *A* = 20kbp we span 80% of the region with 5 haplotypes.

### A short note on haplotype accuracy

While our focus is on the feasibility of generating long haplotypes, accuracy is also an important consideration with next generation technologies that may have undesirable raw read error rates. We used our previously developed tools, HASH, and HapCUT [12,13] to phase haplotypes while accounting for error. The Pacific Biosciences simulator was used to generate reads under realistic error models. The simulator takes a single parameter *ε* as input, reflective of the overall error rate. We chose *ε* ∈ {0.05, 0.1, 0.15}. As our subreads are long, we assumed correct alignment of all reads (Methods).

Many homozygous sites appear heterozygous due to missed base calls. For example, we observe 202K heterozygous sites at *ε* = 0.05 in a region with 936 known SNPs. Using a statistical test for filtering, only 3 of the erroneous sites remain, and none of the ‘true’ SNPs is eliminated. Table 1 summarizes the false negative and false positive rates for *ε* ∈ {0.05,0.10,0.15}, and *c* ∈ {10×, 15×}.

**Table 1:** Filtering Erroneous Heterozygous Sites

Error Rate, Coverage	% of False Positives	% of False Negatives
5%,10×	0	0.467
10%,10×	0	0.197
15%,10×	0	0.142
5%,15×	0	0.329
10%,15×	0	0.149
15%,15×	0	0.114

This table shows false positive rates (% of actual SNPs filtered) and false negative rates (% of erroneous sites not filtered) when sites are filtered using the likelihood ratio with a cutoff at -1.

For *c* = 10×, *ε* ∈ {0.05, 0.1}, we were able to perfectly assemble the haplotypes. Even with *ε* = 0.15, we were able to assemble haplotypes with HER= 2.25%, SER=0.76%. Increasing coverage to *c* = 15×, we achieved HER=1.39%, SER=0.11%. As more data becomes available, we will exploit the error characteristics and related base level quality values to further improve haplotyping accuracy.

## Conclusions

In spite of a long history and success with Sanger sequencing, the feasibility of assembling meaningful haplotypes with next generation sequencing has been questioned. Here, we demonstrate that with a judicious choice of parameters and strobe sequencing, long (and accurate) haplotypes can be effectively generated. The most important parameter appears to be the flexibility in choosing advance lengths, available with strobe sequencing. Even with only *k* = 2 strobes, and coverage *c* = 10×, we can achieve long haplotypes. On the target HLA region, we covered 80% of the region with 5 haplotypes.

Surprisingly, the optimal design for haplotyping heavily favors longer advances, and the trend does not change with higher values of *A, L*, *c*, or number of strobes. Here, we only provide a partial explanation, suggesting that the longer advances level the probability of all edges. A rigorous explanation based on extending the Erdós Renyi theory to the interval-like SNP-Graphs will be the focus of future efforts. Other parameters influence haplotype lengths as well, and our results help determine the optimal values.

Here, we use the ‘number of bp sequenced (coverage)’ as the “cost” of the design, and optimized parameters after fixing coverage. However, other cost factors might be reasonable. For example, it may be more expensive to generate reads with longer inserts. Also, more biological sample is needed (and wasted) with longer inserts, and that can be a limitation when sample is limited (as in tumors). Our simulated annealing software for optimizing parameters can easily be modified to deal with a custom cost function.

Finally, while haplotype assembly can generate long haplotypes, it is not yet capable of separating entire chromosomes. However, other techniques such as chromosome dissection and amplification can generate long scaffolds connecting distal sites. Used in conjunction with Haplotype assembly on strobe sequences, chromosome level haplotyping is indeed feasible, even without familial information.

## Methods

### Data source

The data source was derived from available human assemblies including HuRef [11]. For our simulations we used data from chromosome 1 of the HuRef Genome. While the majority of the experiments were performed on the first 10Mbp of chromosome 1, tests in other regions show similar results. For simulations in the HLA region we used a 3.5Mb interval on chromosome 6.

### Simulator

The input to the simulator is a data source, *D*, containing a list of heterozygous sites and their respective coordinates, and the parameters of the reads (*L*, *c*, *A*, *k*, (*α_i_*, *β_i_*)). The S50, N50, and AN50 metrics are output. The algorithm is described below. It simulates subreads as fixed intervals of size *L/k,* with advances chosen from the appropriate *β*-distributions. The nodes of SNP-Graph are connected by an edge if a fragment overlaps their locations. The procedure GetSummary computes the different metrics at the end of the simulation.

**proc** SIMULATE(*D*, *L*, *c*, *A, k*, (*α*_1_, *β*_1_)*,*.., (*α_k_*_–1_, *β_k_*_–1_))

1. Initialize SNP-Graph by creating a node for each SNP in *D*

2. Set 

3. Repeat *N* times

3.1. Select random start position, *d*_0_; Set *S* = *ϕ*

3.2. for 1 ≤ *i* <*k*

3.2.1 Set advance *a_i_* ← ***D***(*α_i_*, *β_i_*) (* *β*-dist *)

3.2.2 Set *d_i_* = *d_i_*_–1_ + *L/k* + *a_i_*

3.2.3 Add SNPs in intervals [*d_i_*, *d_i_* + *L*/*k*] to *S*

3.3. Add edge (*s_i_*, *s_j_*) and edge (*s_j_*, *s_i_*) to SNP-Graph for all (*s_i_*, *s_j_*) in *S*

4. (*S*50, *N*50, *AN*50) = GETSUMMARY(SNP-Graph)

### Computing optimal (*α, β*)

We used a Simulated Annealing (SA) algorithm to compute the optimal (*α, β*) values. To test the performance of the SA, we also used a slower coarsegrained optimization.

#### Simulated Annealing (SA)

We start with *α, β* chosen at random from the range (0,3.5]. Empirically, Temperature *T* was selected to be 11,000, and reduced by a fixed amount in each iteration. The neighboring solution was selected at random from {(*α* ± *s*, *β*)*,* (*α, β* ± *s*)}. We set *s* = 0.5 for the first half of the iterations, and set *s* = 0.1 for the remaining to allow for finer optimization. This allows for a free exploration of the search space, followed by fine grained optimization at the end. Due to a large variation in AN50 for a fixed (*α*, *β*)*,* we recompute AN50 values for the current solution and the neighbor, making it easier to escape an artificially high value. We maintain a list of all (*α, β*,AN50) triples observed.

**proc** SIMULATEDANNEALING(*D*, *L*, *c*, *A, k*)

1. Initialize grid, *G*

(* *G*(*α*, *β*) is a list of observed *AN*50 values *)

2. Set (*α*, *β*) ← (0, 4] × (0, 4]

3. For all 1 ≤ *i* ≤ *I*

3.1 Set *s* = 0.5 if *i* < I/2; else Set *s* = 0.1

3.2 (α′, *β*′) ← {(*α* ± *s*, *β*)*,* (α, *β* ± s)}

3.3 *G*(*α, β*) = *G*(*α, β*) ;∪ *Simulate*(*D, L, c, A, k, α, β*))

3.4 *G*(*α*′, *β*′) = *G*(α′, *β*′) ∪ *Simulate*(*D, L,* c, *A,* k, *α*′*,* β′))

3.5 AN50 = median(*G*(*α*, *β*))

3.6 AN50’ = median(*G*(*α*′,*β*′))

3.7 Set *T* = *T* – *T*_0_/*I*; ∆ =AN50’-AN50

3.8 Move to (*α*′, *β*′) with probability 

### SA performance

We use an exhaustive coarse-grained optimization to check the performance of SA. Each (*α*, *β*) pair for *α* ∈ (0, 3.4] and *β* ∈ (0 – 3] was chosen with step sizes of 0.2 and 0.1 respectively. For each value, we performed 25 simulations, and recorded the median AN50. We compared SA and coarse grained optimization for *c* = 20×, *L* = 900bp, *A* = 9kbp to match the parameters currently available for strobe sequencing. See Figure 3. The coarse grained optimization entails a total of 12, 750 simulations, each about 1CPU min. on a PC. By contrast, SA achieves a finer grained optimization using only 450 simulations. The results are consistent with the two methods (Additional File 2).

### Calling heterozygous sites (SNPs)

After running our simulated fragments through the Pacific Biosciences error simulator and aligning the erroneous fragments (since our data is simulated, we use original fragments to perfectly align the erroneous fragments), we used statistical methods to differentiate heterozygous sites caused by true SNPs versus those caused by error. If a heterozygous site has a coverage of n (n fragments overlap the site), there are *n*_1_ counts of the dominant allele and *n*_2_ = *n* – *n*_1_ counts of the minor allele.

*H*_0_: The heterozygous site has no bias in the two alleles; the two alleles both have a 50% chance of appearing with a small probability of error.

*H*_1_: The heterozygous site always shows one allele with a small probability of error

Let *ε* be the probability of a miscalled base. Then, the likelihood ratio statistic is given by(2)

The likelihood ratio Λ asymptotically approaches the *χ*^2^ distribution. However, we empirically selected Λ = –1 as the cut-off for calling heterozygous SNPs.

## Authors' contributions

All authors participated in planning the experiments. CL executed the simulations, derived the conclusions and wrote the manuscript. VBansal implemented the simulator, and the metrics. AB helped design and implement the Pacific Biosciences simulator, and consulted on the experiments. VB assisted with writing the manuscript.

## Competing interests

The authors declare that they have no competing interests.

## Supplementary Material

Additional File 1 **Haplotype Accuracy** Example of haplotype edit rate (HER) and switch error rate (SER) Click here for file

Additional File 2 **Contour Plots** Comparison of the contour plots of SA and Coarse grained optimization shows that optimal (*α*, *β*) range of both approaches are similar. Different Metrics (S50, N50, AN50) also produce similar results. Click here for file

Additional File 3 **Simulated Annealing Results for Figure**5 These tables show the optimal AN50 and the (α, *β*) values found by simulated annealing. All the optimal *β*-distributions are similar and skewed towards longer advance lengths. Click here for file
