# Supplementary material for: Strobe sequence design for haplotype assembly
Source: BMC Bioinformatics. 2011 Feb 15;12(Suppl 1):S24. doi: 10.1186/1471-2105-12-S1-S24 (PMC3044279; doi:10.1186/1471-2105-12-S1-S24)
Supplement: Additional File 1 — Haplotype Accuracy Example of haplotype edit rate (HER) and switch error rate (SER) [file 1471-2105-12-S1-S24-S1.pdf]

Figure S1

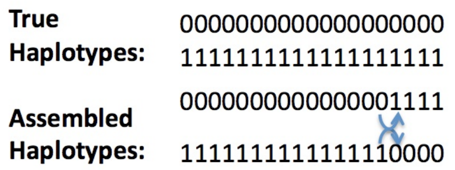

Figure S1: **Haplotype Accuracy**. The haplotype edit rate(HER) in this example, given by the fraction of incorrectly called alleles, is $\frac{4}{19}$ while the switch error rate (SER), defined as the number of crossovers per site required to match the correct haplotype is $\frac{1}{18}$.
