# Supplementary material for: Strobe sequence design for haplotype assembly
Source: BMC Bioinformatics. 2011 Feb 15;12(Suppl 1):S24. doi: 10.1186/1471-2105-12-S1-S24 (PMC3044279; doi:10.1186/1471-2105-12-S1-S24)
Supplement: Additional File 2 — Contour Plots Comparison of the contour plots of SA and Coarse grained optimization shows that optimal (α, β) range of both approaches are similar. Different Metrics (S50, N50, AN50) also produce similar results. [file 1471-2105-12-S1-S24-S2.pdf]

Figure S2

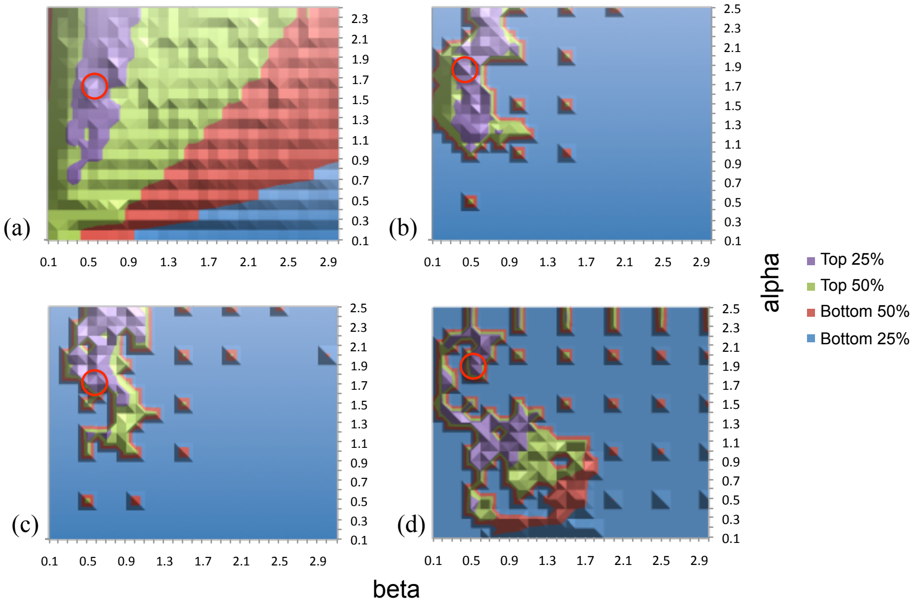

Figure S2: **Contour Plots**. Comparison of the contour plot of SA and Coarse grained optimization shows that optimal (α, β) range of both approaches are similar. Different metrics (S50, N50, AN50) also produce similar results. (a) Coarse grain optimization for AN50 (b) SA contour for AN50 (c) SA contour of N50 (d) SA Contour of S50. Optimal (α, β) value for each is circled. All simulations were performed on the first 10Mbp of chr1 (HuRef) using $L = 900$bp, $c = 20\times$, $A = 9$kbp.
