# Supplementary material for: Strobe sequence design for haplotype assembly
Source: BMC Bioinformatics. 2011 Feb 15;12(Suppl 1):S24. doi: 10.1186/1471-2105-12-S1-S24 (PMC3044279; doi:10.1186/1471-2105-12-S1-S24)
Supplement: Additional File 3 — Simulated Annealing Results for Figure5 These tables show the optimal AN50 and the (α, β) values found by simulated annealing. All the optimal β-distributions are similar and skewed towards longer advance lengths. [file 1471-2105-12-S1-S24-S3.pdf]

Table S1 - Simulated Annealing Results for Figure 5

These tables shows the optimal AN50 and the (α, β) values found by simulated annealing. All the optimal β-distributions are similar and skewed towards longer advance lengths.

Table S1a: **AN50 and Optimal** (α, β) **for Figure ??a**

Max Adv Len (kbp)	AN50	(α, β)
9	161607	(1.9,0.7)
20	900068	(3.8,0.8)
30	2804943	(2.6,0.6)
40	4389053	(2.8,1.0)
50	10658401	(3.6,0.7)

Table S1b: **AN50 and Optimal** (α, β) **for Figure ??b**

Coverage	AN50	(α, β)
10	84816	(2.6,1.0)
20	152625	(1.7,0.6)
30	158652	(2.1,0.7)
40	166004	(3.1,1.0)
50	195282	(1.6,0.5)
60	226540	(1.1,0.4)
70	226540	(1.8,0.9)
80	226540	(3.6,0.6)
90	226540	(3.2,0.9)

Table S1c: **AN50 and Optimal** (α, β) **for Figure ??c**

Read Length	AN50	(α, β)
300	60593	(2.7,0.8)
500	117657	(3.3,0.7)
700	133155	(2.4,0.8)
900	144690	(0.9,0.5)
1100	155397	(2.3,0.6)
1300	157248	(2.5,0.6)
1500	158652	(1.4,0.4)
2000	166004	(1.4,0.5)
4000	166004	(3.1,0.5)

Table S1d: **AN50 and Optimal** (α, β) **for Figure ??d**

Num of Strobes (kbp)	AN50	$(\alpha_1, \beta_1), ...(\alpha_k, \beta_k)$
2	88084	(1.8, 0.4)
3	108110	(2.7, 0.3), (2.0, 1.6)
4	110740	(2.9,0.1), (2.9, 1.0), (0.3, 0.5)
5	93870	(3.5, 0.1) (2.7, 0 .3), (2.7, 2.0), (0.8, 1.6)
6	86836	(3.1, 0.6), (3.2, 1.1), (2.9, 1.6), (2.6, 2.5), (2.5, 2.5)
